# Impact of l-Arginine Metabolism on Immune Response and Anticancer Immunotherapy

**DOI:** 10.3389/fonc.2018.00067

**Published:** 2018-03-16

**Authors:** Sun-Hee Kim, Jason Roszik, Elizabeth A. Grimm, Suhendan Ekmekcioglu

**Affiliations:** ^1^Department of Melanoma Medical Oncology, The University of Texas MD Anderson Cancer Center, Houston, TX, United States

**Keywords:** arginine, NO synthase, arginase-1, cyclooxygenase-2, microsomal prostaglandin E synthase-1, immunotherapy, metabolism, immune response

## Abstract

The progression from neoplastic initiation to malignancy happens in part because of the failure of immune surveillance. Cancer cells successfully escape immune recognition and elimination and create an immune-suppressive microenvironment. A suppressive metabolic microenvironment may also contribute to ineffective T-cell function. Tumor progression is characterized by a complex network of interactions among different cell types that cooperatively exploit metabolic reprogramming. As we start to recognize that cancer cells use different metabolism processes than normal cells do, a better understanding of the functional mechanisms of the regulation and reprogramming of the metabolic landscape in cancer cells is crucial to successful immunotherapy strategies. However, the exact role of metabolism in T cells and in the tumor microenvironment is not known. One pathway that plays an important role in the regulation of immune cell reactivity is arginine metabolism, which has complex cellular functions. l-arginine and its downstream metabolites (e.g., ornithine and citrulline) could be essential to T-cell activation and thus modulate innate and adaptive immunity to further promote tumor survival and growth. Identifying metabolic targets that mediate immunosuppression and are fundamental to sustaining tumor growth is key to increasing the efficacy of immunotherapies.

## Introduction

Metabolic pathways and their intermediates have been shown to contribute to the formation of the immunosuppressive tumor features that exclude immune cells from the tumor microenvironment or impair their ability to respond. Manipulating these metabolic changes can strengthen antitumor immune responses by recovering the functions of the T cells. Thus, cancer immunotherapy strategies focus on metabolism to promote immune cells’ acquisition of sufficient metabolites to preserve their antitumor responses.

Tumor progression is characterized by a complex network of communication among different cell types and cancer cells that stimulates the metabolic reprogramming of the cancer cells and thus influences their functionality. Our research suggests that deleterious tumor inflammatory processes occur in response to microenvironmental communications and are driven by intrinsic inflammatory processes (1). Much recent interest and new immune system data suggests that cellular bioenergy links metabolism with inflammation and immunity to protect normal cells from immune death (i.e., checkpoint inhibition) and to restore homeostasis with minimal tissue damage (2), and provides a complex balance to make it beneficial for the host. However, cancer cells with these self-protective mechanisms become resistant to immune attack. Clinically evident cancers are those that have evaded the immune response. Inflammation and immune evasion are hallmarks of cancer progression, as evidenced by the fact that cancer patients with fewer immune cells in their biopsied tissues have poorer responses to immunotherapies (3). These cells may include the direct involvement of immune cells, including macrophages, T cells, B cells, natural killer cells, dendritic cells, and myeloid-derived suppressor cells (MDSCs) (4).

Some of the mechanisms the immune system uses to fight infectious diseases also promote immune suppression in the tumor microenvironment. Tumor resistance to the immune response is assisted by the immunoediting of the immune signals induced by the tumors and their metabolites (2), possibly including that of checkpoint inhibitor upregulation, *via* inflammatory mechanisms mediated by arginine metabolism (5). This review presents the role of arginine metabolism in the major inflammatory processes that we have found to drive the survival of melanoma and other cancers, including those processes underlying these tumors’ resistance to therapy, particularly immunotherapy.

## Arginine Metabolism as a Major Requirement for Inflammatory Networks and Products

Human tumors support an immunosuppressive microenvironment that often prevents effective immunotherapy. Because of their effectiveness and the long-term responses they elicit, cancer immunotherapies have changed the landscape of cancer care. Despite the major breakthroughs in cancer immunotherapy of the past decade, the success to date does not meet the promise of the approach. Different physiological mechanisms can prevent durable responses to immunotherapy. Some of these mechanisms have been characterized molecularly and may be triggered by the tumor microenvironment, resulting in impaired immune effector cell function.

**Figure 1:**
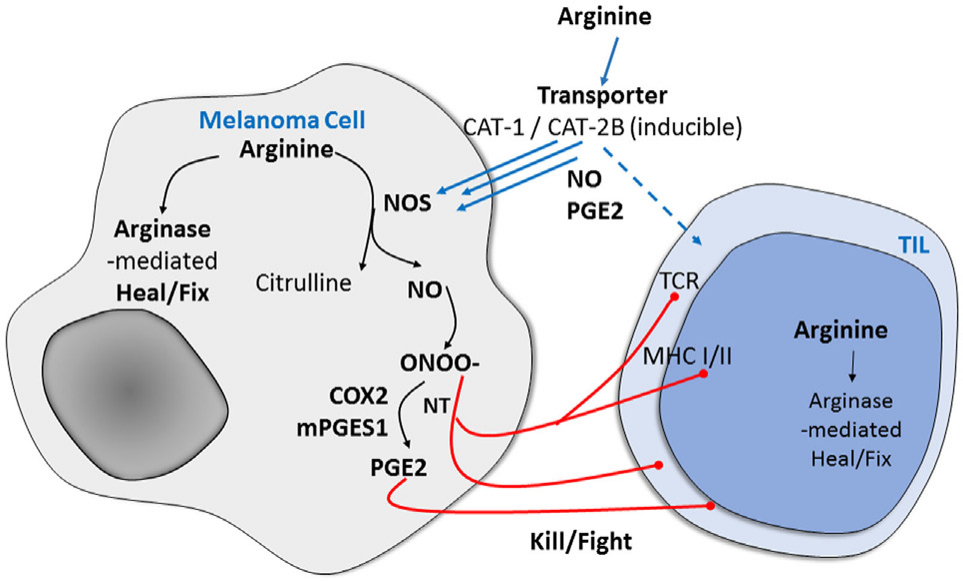
Potential mechanism of arginine metabolism in cancer cells versus immune-infiltrating cells in human melanoma. After arginine enters mammalian cells through the membrane-bound transporters CAT1 and CAT2B, it is metabolized by one of the NO synthase (NOS) enzymes to produce nitric oxide (NO), which is associated with immune suppression and results in the expression of specific markers and functional changes.

Arginine metabolism is one of the mechanisms responsible for tumor progression and it is highly compartmentalized due to expression of enzymes involved in arginine metabolism in various cells. l-arginine is a multipurpose amino acid that also serves as a precursor for multiple metabolites, including polyamines and nitric oxide (NO), which have strong immunomodulatory properties (Figure 1). l-arginine is the substrate for four enzymes, several of which exist as multiple isoforms: NO synthases (NOSs), arginases (ARGs), glycine aminidotransferase, and l-arginine decarboxylase (6). To encounter the enzymes involved in its metabolism, l-arginine must be transported through the plasma membrane *via* cationic amino acid transporters (CATs) and metabolized by NOS enzymes (7). Using the Cancer Cell Line Encyclopedia database, we found that CAT2B expression varies among human melanoma cells and that a subset of human melanoma cells has a very high level of CAT2B expression, but that CAT1 expression is similar among all the cell lines. CAT1 is constitutively expressed in most tissues, whereas CAT2B is cytokine inducible (8). Thus, we speculate that the diverse expression of CAT2B may be associated with the inflammatory factors expressed in human melanoma cells.

After arginine enters mammalian cells through the membrane-bound transporters CAT1 and CAT2B, it is metabolized by one of the NOS enzymes. The enzymatic production of NO is cell-type specific, with cytokine-driven inducible NOS (iNOS) noted initially for the burst of higher levels as part of the pathogen defense system. Neuronal cells use neuronal NOS to produce NO for signaling. The third NOS, endothelial NOS, regulates NO production in endothelia and is responsible for vascular relaxation (9). In previous studies, we found that the selectively increased metabolism of l-arginine in melanoma and other human tumors is associated with immune suppression and neovascularization and that the arginine metabolic product of NO results in the expression of specific markers of tumor progression and in functional changes (10, 11). We and others have also found that increased arginine metabolism in melanoma tumors and their microenvironment mediates a previously unappreciated system that is fundamental to sustaining the growth of many cancers while specifically causing immunosuppression (11–14).

The metabolism of arginine by a family of NOSs generates NO, which can affect molecular pathways in melanoma cells and the melanoma microenvironment by defined posttranslational modifications. NO is a relatively stable intracellular molecule that diffuses through lipid bilayers and persists for at least 7 s (15). NO can react with superoxide to form peroxynitrite, which rapidly forms two posttranslational modifications on proteins, nitrosylation of thiols and nitration of tyrosines (NT) (16, 17). A second arginine metabolism *via* ARG leads to proline and polyamine production, which may also contribute to tumor progression. Therefore, overall arginine metabolism depends on the activity of the NOS and ARG enzyme families. NOS oxidizes arginine to citrulline and NO, and ARG hydrolyzes arginine into ornithine and urea. We and others have demonstrated that many human tumors, including melanoma, express iNOS (18–23). iNOS expression and NO production have been shown to promote many cancers’ growth, survival, and resistance to therapy. One recent study of the genomic and transcriptomic features of the response of metastatic melanoma to anti-programmed cell death protein 1 (PD-1) therapy revealed that a transcriptional signature is related to innate anti-PD-1 resistance (24). Using the same clinical dataset, we found that iNOS expression in metastatic melanoma is associated with response to anti-PD-1 therapy (unpublished data). Therefore, understanding the regulation of immune cell response and functions of tumor-expressed iNOS will provide significant opportunities for therapeutic intervention. ARG-1 activity is associated with polarized, protumoral M2 tumor-associated macrophages (25). Suppression of T-cell activation, proliferation, and differentiation by macrophage ARG-1 dominated by M2 macrophages and interactions between macrophage metabolism and M1/M2 polarization is an urgent matter to be resolved in cancer. Together, ARG and NOS enzymes metabolize arginine and are critical components of immune suppression pathways, and the metabolic products of these enzymes are imperative mediators of T-cell function in cancer (12).

The importance of arginine metabolism as a novel field of investigation includes that of arginine depletion retarding the growth of some cancers (26), whereas others report that arginine supplementation enhances antitumor effects, probably by enhancing immune function (11, 27). Arginine supplementation has been discovered to stimulate T cell and natural killer cell activity and promotes the production of pro-inflammatory cytokines (28, 29). The roles of arginine in the context of NOS and ARG activity and in tumor-derived NO need to be understood.

## Arginine Metabolism and NOS and Cyclooxygenase

Preliminary data from our laboratory and others (24, 25) indicate that NO activates cyclooxygenase-2 (COX-2) and other inflammatory mediators, thereby creating a pro-oxidant microenvironment that supports cancer cell growth and suppresses antitumor immunity. iNOS/NO positively regulates the production of COX-2, microsomal prostaglandin E synthase-1 (mPGES1), and prostaglandin E2 (PGE2). COX-2 and iNOS can be produced concurrently in several inflammatory conditions (24). In our previous study, a tissue microarray analysis of stage III melanoma showed that most mPGES1-positive tissues were also positive for iNOS and had co-localized mPGES1 and iNOS expression. n addition, transient iNOS expression and NO donors dramatically enhanced PGE2 production, suggesting that iNOS and NO are upstream of PGE2 biosynthesis in melanoma cells. We found that mPGES1 was tyrosine-nitrated by NO. Our data suggest that iNOS regulates mPGES1 activity through a posttranslational NT modification, which ultimately enhances PGE2 production; that iNOS activates the mPGES1/PGE2 pathway; and that the cross talk between iNOS and mPGES1 promotes inflammation, which favors melanoma progression (24).

In melanoma and other cancers, PGE2 plays a role in immune responses, acting as both a pro-inflammatory mediator and a potent immune suppressant (26). In melanoma, specifically, tumor-derived COX activity is the key suppressor of type I interferon-mediated tumor elimination and induces an inflammatory signature associated with progression (27). The PGE2-mediated stimulation of immunosuppressive cells, including T regulatory cells (Tregs) and MDSCs, may also be an important mechanism of immune suppression. PGE2 has an important role in redirecting the differentiation of human dendritic cells into MDSCs, and the inhibition of COX-2 or PGE2 receptors abolishes MDSCs’ functions and their CXCR4-CXCL12-mediated attraction to the cancer environment (28). PGE2 stimulates the *de novo* conversion of Tregs from naïve CD4+ T cells (29), and Tregs expressing PGE2 receptors are preferentially recruited to factors expressed by COX-2-expressing tumor cells (30). Elevated MDSCs and Tregs suppress T-cell infiltration into the tumor site and/or inhibit T-cell activity. In a previous study of stage III melanoma, our tissue microarray analysis demonstrated that high mPGES1 staining intensity was significantly associated with low CD8 levels, and we found that patients whose tumors had a high-mPGES1, low-CD8 expression signature had a significantly increased risk of death (31).

Several recent reports suggest that targeting PGE2 metabolism could help reduce programmed death-ligand 1 (PD-L1)-mediated immune suppression (27, 32, 33). One study showed that COX-2 and PD-L1 were expressed in both primary melanoma lesions and non-matching lymph node metastases and that the inhibition of COX-2 activity by celecoxib downregulated PD-L1 expression in both BRAFV600E A375 and NRASQ61R SK-MEL-2 melanoma cell lines (32). In another study, an analysis of murine bone marrow cells cocultured with murine MBT-2 bladder tumor cells demonstrated that the COX-2/mPGES1/PGE2 pathway regulates PD-L1 expression in tumor-associated macrophages and MDSCs (33). A third study showed that conventional nonsteroidal anti-inflammatory drugs and COX-2 inhibitors combined with an anti-PD-1 monoclonal antibody promoted a much more rapid regression of mouse melanoma tumors than the anti-PD-1 antibody alone did (27). This result suggests that the COX-2/mPGES1/PGE2 pathway contributes to immune evasion and that nonsteroidal anti-inflammatory drugs and COX-2 inhibitors are useful adjuvants to immune-based anticancer therapies.

Our data show not only the NO-mediated modulation of tumor-infiltrating leukocyte (TIL) growth but also the COX-2-mediated production of PGE2 in human melanoma. Other potential sources of NO from arginine metabolism in cancers include MDSCs, which are a heterogeneous population of myeloid cells that often infiltrate cancers and sites of inflammation and infection and have a remarkable ability to suppress T-cell responses (30). We have previously reported that iNOS expression in melanoma is significantly associated with apoptosis resistance and shorter patient survival (18, 31). We recently observed global NT expression in stage IIIc and IV melanomas in humans. NT is a marker of NO’s reaction with reactive oxygen species to form peroxynitrite, which irreversibly nitrates tyrosines. NT expression in tissues is associated with poor TIL growth and poorer response to TIL-based therapy. Protein-associated NT has been identified as a surrogate marker of *in situ* inflammation, but its role in cellular immune response has not been studied broadly. One intriguing study showed that the oxidation of tyrosine to NT on either T-cell receptor or major histocompatibility complex (MHC) molecules can disturb CD8 T cells’ recognition of the MHC class I-restricted epitope of lymphocytic choriomeningitis virus glycoprotein (32). This study also demonstrated that T cells’ specific recognition of nitrated epitopes is not limited to MHC class II epitopes but that the conversion of tyrosine to nitrotyrosine can also intensely disturb these cells’ recognition of MHC class I-restricted epitopes. If, like CD4 T cells, CD8 T cells can recognize epitopes containing an NT posttranslational modification as antigenic, this would certainly affect TIL activation in melanoma patients.

## Conclusion

Following the success of checkpoint inhibitors in many tumor types, immunotherapy has become a major focus of cancer researchers and clinicians. Our mission is to guide oncology research by improving our understanding of the role of tumors’ interactions with their microenvironment and with immune cells functions in particular. Arginine metabolism is one of the mechanisms responsible for immune response to tumor progression. Because different cells express enzymes involved in the process, arginine metabolism is highly dysregulated in cancer. The concept of antitumor immune response, based on usage of arginine *via* NOS and ARG, is timely topic to focus on to set our knowledge regarding their role in current immunotherapy approaches. The extent to which immune cells and tumor cells in the tumor microenvironment respond to arginine metabolism could predict better immunotherapy outcomes.

## Author Contributions

All authors (S-HK, JR, EG, and SE) were involved in building the concept and participated in the writing based on their expertise area. S-HK was involved mainly in writing; JR was involved in concept and analyses, EG was involved in sharing her expertise, reviewing, and editing; and SE was mainly involved in writing, concept design, reviewing, and editing.

## Conflict of Interest Statement

The authors declare that the research was conducted in the absence of any commercial or financial relationships that could be construed as a potential conflict of interest.
